# Patient Adherence to Oral Anticancer Agents: A Mapping Review of Supportive Interventions

**DOI:** 10.3390/curroncol30120744

**Published:** 2023-11-30

**Authors:** Saima Ahmed, Carmen G. Loiselle

**Affiliations:** 1 Division of Experimental Medicine, Faculty of Medicine and Health Sciences, McGill University, Montreal, QC H4A 3J1, Canada; saima.ahmed2@mail.mcgill.ca; 2 Segal Cancer Centre, CIUSSS du Centre-Ouest-de l’Île-de Montréal, Montreal, QC H3T 1E2, Canada; 3 Ingram School of Nursing, Faculty of Medicine and Health Sciences, McGill University, Montreal, QC H3A 2M7, Canada; 4 Department of Oncology, Faculty of Medicine and Health Sciences, McGill University, Montreal, QC H4A 3T2, Canada

**Keywords:** oral anticancer agents, medication adherence, supportive intervention, knowledge synthesis, mapping review

## Abstract

The development and use of oral anticancer agents (OAAs) continue to grow, and supporting individuals on OAAs is now a priority as they find themselves taking these drugs at home with little professional guidance. This mapping review provides an overview of the current evidence concerning OAA-supportive adherence interventions, identifying potential gaps, and making recommendations to guide future work. Four large databases and the grey literature were searched for publications from 2010 to 2022. Quantitative, qualitative, mixed-method, theses/dissertations, reports, and abstracts were included, whereas protocols and reviews were excluded. Duplicates were removed, and the remaining publications were screened by title and abstract. Full-text publications were assessed and those meeting the inclusion criteria were retained. Data extracted included the year of publication, theoretical underpinnings, study design, targeted patients, sample size, intervention type, and primary outcome(s). 3175 publications were screened, with 435 fully read. Of these, 314 were excluded with 120 retained. Of the 120 publications, 39.2% (*n* = 47) were observational studies, 38.3% (*n* = 46) were quasi-experimental, and 16.7% (*n* = 20) were experimental. Only 17.5% (*n* = 21) were theory-based. Despite the known efficacy of multi-modal interventions, 63.7% (*n* = 76) contained one or two modalities, 33.3% (*n* = 40) included 3, and 3.3% (*n* = 4) contained four types of modalities. Medication adherence was measured primarily through self-report (*n* = 31) or chart review/pharmacy refills (*n* = 28). Given the importance of patient tailored interventions, future work should test whether having four intervention modalities (behavioral, educational, medical, and technological) guided by theory can optimize OAA-related outcomes.

## 1. Introduction

Driven by ease of administration, patient convenience, potential cost-effectiveness, and enhanced quality of life, oral anticancer agents (OAAs) are being rapidly developed, tested, and approved for patient use. Able to be taken by patients at home rather than in the hospital, OAA use increased substantially during the COVID-19 pandemic, as drugs that can be taken at home, orally, were favored over intravenous ones when possible [1,2]. Currently, OAAs represent half of all cancer drugs sold in Canada [3] and 50–60% of the global oncology drugs in the development pipeline [4].

Compared to intravenous (IV) chemotherapy, OAAs refer to pills or tablets taken by mouth to treat cancer and are classified under three categories: (1) traditional agents, a systemic approach taking advantage of the rapid division of cancer cells to kill all cells within certain phases of the cell cycle (e.g., Capecitabine), (2) targeted agents, referred to as “precision medicines” that are genetically driven, inhibiting a specific pathway in the cancer cell (e.g., Imatinib or Ribociclib), and (3) hormonal agents, manipulating the hormones in cancer cells that require them to grow (e.g., Anastrozole) [5].

The transfer from in-hospital to home-based OAA treatment presents a paradigm shift in cancer care, with the administration and management of treatment now falling to the patient and informal caregivers rather than within the healthcare team. For OAAs to be as effective as possible, patients must follow best practices (e.g., dosage, timing, foods to avoid, etc.), as well as manage their treatment, side effects, and be alert for toxicities. Medication adherence is the primary determinant of treatment success [6], whereby medication-related behaviors “correspond with agreed upon recommendations from their healthcare provider” [7]. In addition to taking medication as prescribed at the same time every day—as alterations in dose and timing can affect treatment outcomes [8]—adherence also includes obtaining the prescription(s), medication initiation and continuation, refills, and following medical recommendations related to medication intake, side effects, and potential complications [9]. However, medication adherence can be challenging [6], with studies reporting OAA adherence ranging from 46 to 100% [10]. Lower than prescribed adherence can cause reduced medication effectiveness, increased healthcare service use, and higher costs, such as more medical visits, higher hospitalization rates, longer hospital stays, and possibly lower survival [11,12,13,14].

A review of factors influencing non-adherence to OAAs [15], for instance, identified three modifiable factors across studies that interventions may focus on: side effects and toxicities, forgetfulness, and the provision of reliable information. As OAAs have grown exponentially in popularity, supportive interventions promoting medication adherence have been developed and tested. Published studies vary in scope in terms of participant profiles, OAA types, intervention theoretical underpinnings and modalities, study design, outcomes of interest, as well as operationalization of the medication adherence construct. While systematic, meta, and scoping reviews have been published, and offer in-depth analyses and specificity on the evidence on this topic [16,17,18], there remains a need for a wider-scope appraisal of the OAA literature in terms of study type, design, and intervention quality, as well as outcomes of interest. A mapping review provides a useful structured overview of relevant research by “categorizing, classifying, characterizing patterns, trends or themes in evidence production or publication” [19,20]. Despite increased interest and a growing body of literature on this topic, a mapping review has never been conducted to assess gaps for future work to address. Cataloguing relevant information into domains, such as theoretical underpinnings, targeted samples, and outcomes of interest, can provide further insights into broader questions, such as, what work has been done so far, and what needs to be done in the future [21,22]. Using visual displays to summarize data makes similarities and differences among studies clearer while explicitly addressing potential issues [21].

## 2. Objectives

The main objectives of this mapping review are to identify, describe, and provide an overview of the body of literature on supportive interventions promoting OAA adherence, and identify gaps and avenues for future work. The recommended evidence-based PICO model, used to formulate a clinical question [23], guided the search strategy for this review, as presented in Table 1.

## 3. Methods

The reporting of this mapping review follows the Preferred Reporting Items for Systematic Reviews and Meta-Analysis (PRISMA) guidelines [24,25].

### 3.1. Search Strategy and Eligibility Criteria

PubMed/Medline, EMBASE, CINAHL, PsycINFO, and the grey literature were searched for articles published between January 2010 and December 2022. An initial search strategy of Medical Subject Headings (MeSH), key text word(s), or text phrases was formulated. Upon consultation, the university-based librarian, an expert in reviews specialized in medicine, helped further strategize the terms used in the search. The finalized search strategy carried out by the first author included: antineoplastic agents, administration oral, oral (chemotherapy* or anti?neoplastic or anti?tumo?r,) neoplasms/or carcinoma/or (cancer* or tumo?r* or carcinoma* or adenocarcinoma* or neoplas* or oncolog* or metasta* or maglignan* or choriocarcinoma* or sarcoma* or lymphoma* or melanoma* or myeloma*), cancer oral* (agent* or medication* or medicine* or therap* or treatment* or drug* or deliver*), oral oncolytic*, medication adherence or patient compliance or health behavior or attitude to health or adherence or compliance or self-efficacy, patient (complian* or activat* or engage* or empower*), health behevio*r.

Quantitative, qualitative, mixed-method studies, theses/dissertations, reports, and conference abstracts were included. Study protocols and review publications were excluded to avoid duplicate data (e.g., separately published protocols and results).

### 3.2. Study Selection and Review

A member of the research team first identified publications by title for their relevance to the review topic and publication eligibility (i.e., quantitative, qualitative, mixed-method studies, theses/dissertations, reports, or conference abstracts). The retained publications were then screened by their abstracts. Once duplicates were removed, those with all inclusion criteria were retained for full-text review and synthesis.

### 3.3. Data Extraction

This mapping review provides a visual overview of the existing evidence related to the topic by coding, cataloguing, and describing the evidence. For each publication, data were extracted using the following categories: (1) year of publication, (2) conceptual or theoretical framework used for study and/or intervention, (3) design, (4) sample, (5) sample size, (6) intervention type and modalities, and (7) primary outcome(s).

## 4. Results

**Figure 1:**
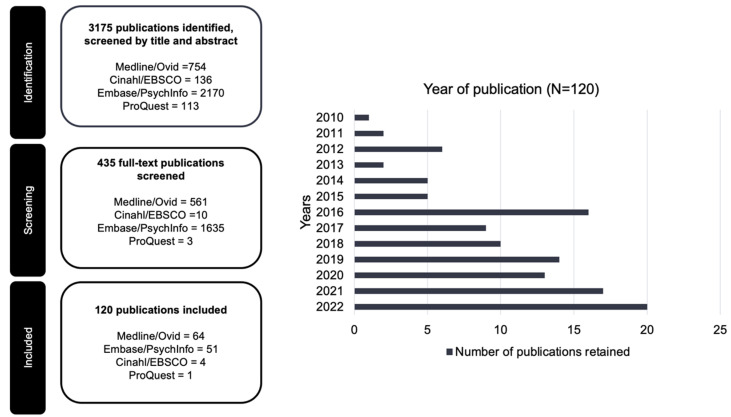
PRISMA flowchart of the publication selection process and years of publication (N = 120).

Here, 3175 publications were initially identified and screened by the title and abstract. Of these, 435 were kept for full-text review, and following this, 314 were excluded as duplicates or for not being relevant or meeting the inclusion criteria. A final sample of 120 publications was retained. The PRISMA flow chart (Figure 1) summarizes the study selection process in detail. A list of all included 120 publications can be found in the Supplementary Material.

### 4.1. Year of Publication

Publications from January 2010 to December 2022 were retrieved. Nearly 82% (*n* = 99) were published from 2016 onward. The year of publication is illustrated in Figure 1 as: 2010 (*n* = 1, 0.8%), 2011 (*n* = 2, 1.7%), 2012 (*n* = 6, 5%), 2013 (*n* = 2, 1.7%), 2014 (*n* = 5, 4.2%), 2015 (*n* = 5, 4.2%), 2016 (*n* = 16, 13.3%), 2017 (*n* = 9, 7.5%), 2018 (*n* = 10, 8.3%), 2019 (*n* = 14, 11.7%), 2020 (*n* = 13, 10.8%), 2021 (*n* = 17, 14.2%), and 2022 (*n* = 20, 16.7%).

### 4.2. Conceptual or Theoretical Framework

Of the studies and/or interventions reported, 17.5% (*n* = 21) were explicitly theory-based, with the Plan-Do-Study-Act (PDSA) model (*n* = 4), self-efficacy (*n* = 3), motivational interviewing (*n* = 2), and one intervention using motivational interviewing to increase self-efficacy (*n* = 1). Table 2 presents a summary of the theories and frameworks used to inform the interventions of the retained publications.

### 4.3. Study Design

**Figure 2:**
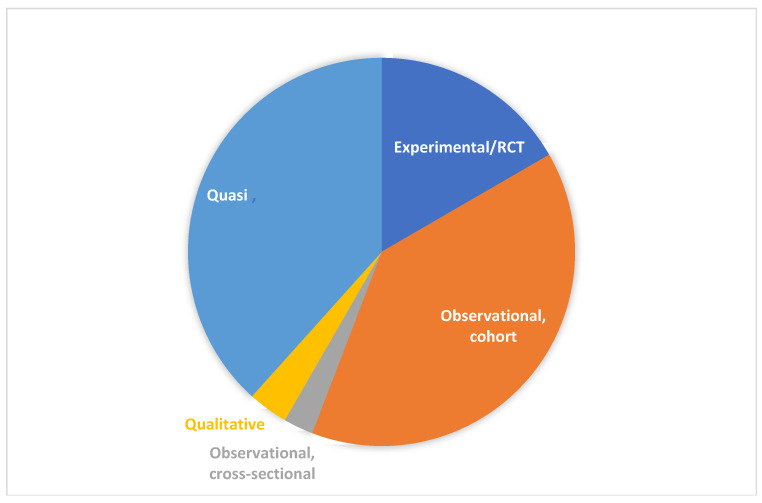
Pie chart of the study design of the retained publications (N = 120).

Among the publications, 39.2% (*n* = 47) used observational cohort designs, 38.3% (*n* = 46) were quasi-experimental, and 16.7% (*n* = 20) were experimental, such as randomized controlled trials (RCTs) or pilot RCTs. Only 3.3% (*n* = 4) relied on qualitative designs and 2.5% (*n* = 3) were observational cross-sectional studies (Figure 2 and Table 3)

### 4.4. Study Sample

Most of the publications (*n* = 89, 75%) used samples with participants diagnosed with various cancers (on OAAs). Only a few publications focused on a particular cancer diagnosis, such as hematological (*n* = 12, 10%), gastrointestinal (*n* = 10, 8.3%), breast (*n* = 5, 4.2%), lung (*n* = 3, 2.5%), or prostate cancer (*n* = 1, 0.8%).

**Figure 3:**
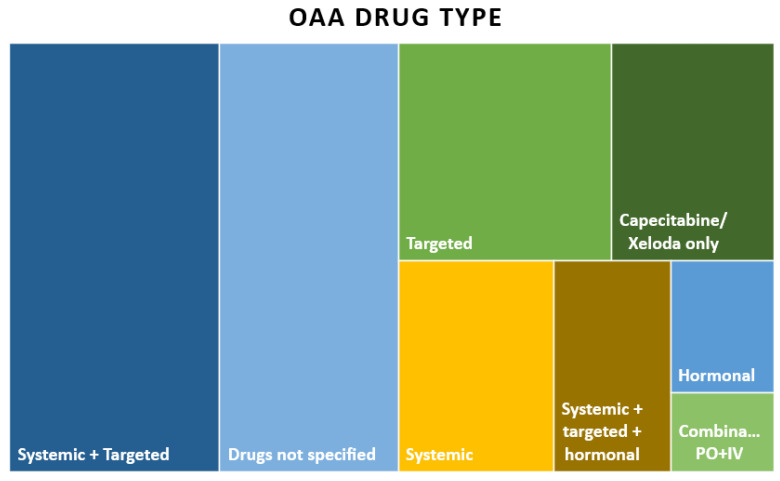
Tree map chart of OAA types (N = 120).

In terms of OAA type, systemic and targeted drugs together were the most common (*n* = 33, 27.5%), followed by targeted only (*n* = 17, 14.2%), Capecitabine/Xeloda only (*n* = 13, 10.8%), mixed systemic therapy (*n* = 12, 10%), all three together—systemic, targeted, and hormonal (*n* = 9, 7.5%), hormonal only (*n* = 5, 4.2%), and a combination treatment of IV and PO (*n* = 3, 2.5%). Of note, 23.3% (*n* = 28) of the publications did not specify OAA drug(s) taken by patients in their study. The distinct study population OAA types are illustrated in Figure 3 and Table 4.

### 4.5. Sample Size

Sample sizes varied across studies, with more than half (62.5%; *n* = 75) having less than 100 participants. Sample sizes were categorized as follows: 1–10 (*n* = 3, 2.5%), 11–30 (*n* = 25, 20.8%), 31–50 (*n* = 18, 15%), 51–100 (*n* = 29, 24.2%), 101–150 (*n* = 13, 10.8%), 151–200 (*n* = 7, 5.8%), 201–250 (*n* = 4, 3.3%), 251–300 (*n* = 7, 5.8%), 301–350 (*n* = 2, 1.7%), more than 400 (*n* = 10, 8.3%), and not specified (*n* = 2, 1.7%).

### 4.6. Intervention Type and Approach

**Figure 4:**
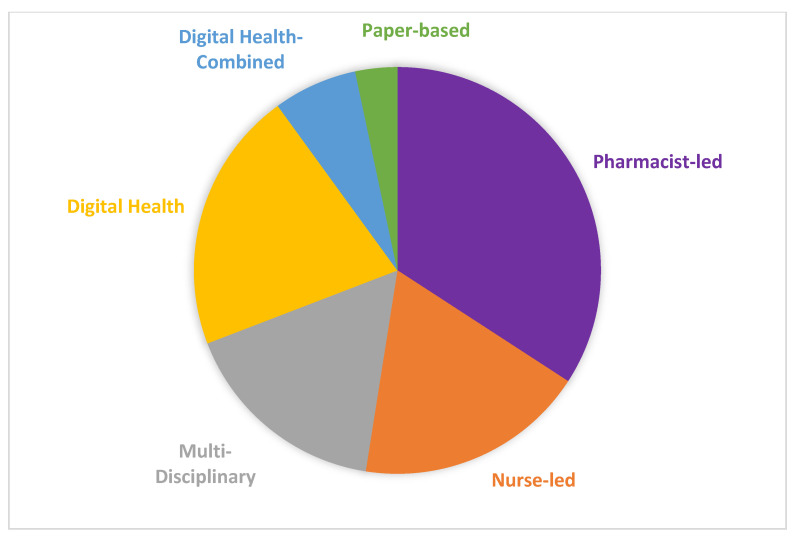
Pie chart of intervention modalities (N = 120).

Interventions that were professionally led were at the forefront (*n* = 83, 69.2%), with pharmacists (*n* = 41, 34.2%), nurses (*n* = 22, 18.3%), and multiple disciplines involved (doctor, nurse, and/or pharmacist combined; *n* = 20, 16.6%). In addition, 20.8% (*n* = 25) were digitally based, 6.7% (*n* = 8) were digital health/healthcare provider combined, and 3.3% (*n* = 4) were paper-based. Figure 4 and Table 5 summarizes the intervention types.

**Figure 5:**
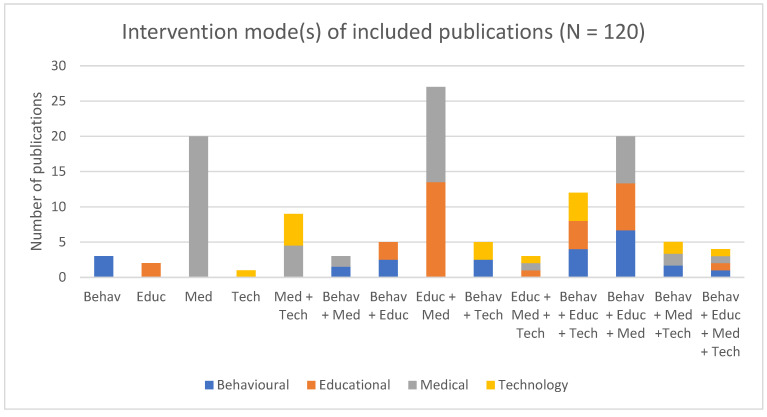
Interventions classified by medical, behavioral, educational, and/or technological approaches (N = 120).

Interventions were further classified into four main approaches: medical—any act with preventative, diagnostic, therapeutic, or rehabilitative claims carried out by a physician or healthcare provider [26]; educational—the provision of information to increase knowledge and skills to help patients better manage their treatment [27]; behavioral—“designed to affect the actions that individuals take” [28]; and/or technological—relying on the internet and/or smartphones [29]. About one-quarter were medical and educational (*n* = 27, 22.5%), followed by medical only (*n* = 20, 16.7%), medical, educational, and behavioral (*n* = 20, 16.7%), and medical and technological (*n* = 9, 7.5%). Of note, only 3% (*n* = 4) of studies contained all four approaches. Figure 5 presents a summary of the interventions by the prevalence of approaches.

### 4.7. Primary Outcome(s)

Most publications had multiple outcomes of interest, and these included (not mutually exclusive) medication adherence (*n* = 74); side effects, toxicities, or adverse events (*n* = 29); number of consults, interventions, or healthcare service use (*n* = 22); knowledge and/or understanding (*n* = 13); quality of life (QoL or HRQoL, *n* = 12); satisfaction and/or perceptions (*n* = 11); and self-efficacy (*n* = 6). A summary of all publications included (*n* = 120) and the corresponding outcome(s) for each is available in the Supplementary Material.

**Figure 6:**
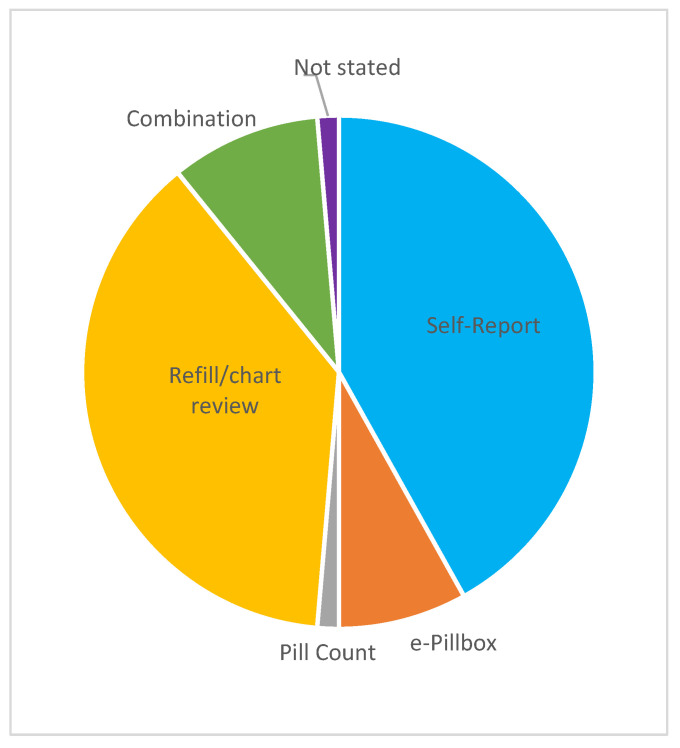
Pie chart of measurement methods for OAA adherence (*n* = 74).

Medication adherence, more specifically, was measured through self-report (*n* = 31), pill count (*n* = 1), electronic/smart pillboxes (*n* = 6), chart review/pharmacy refills (*n* = 28), or a combination of two or more measures (*n* = 7). Figure 6 depicts methods of measuring adherence, while Table 6 further elaborates on the measures used, including self-report, chart refill, and combined methods.

## 5. Discussion

This mapping review sought to identify, describe, and provide an overview of the body of literature on supportive interventions related to OAAs. To our knowledge, this is a first in providing a comprehensive review and visual depiction of publication findings on the topic.

The current state of the literature on OAAs is congruent with their rise in popularity over the past few years, with the greater part of publications produced between 2016 and 2022. This reflects the approval timeframe of commonly prescribed oral cancer drugs and their subsequent rapid uptake as routine cancer treatment [30,31,32].

Taken together, findings from this mapping review emphasize the importance of well-thought-out intervention development, testing, and implementation, as well as transparent reporting of OAA-related outcomes [33,34]. As multiple factors contribute to optimal adherence, single-mode interventions are not found to be as effective as multi-modal interventions (studied among people with chronic conditions) [16,35,36,37].

In addition, interventions should ideally follow established theoretical models or frameworks addressing behavioral factors and underlying processes involved [38,39]. Whereas several studies herein contained a behavioral component, only 17.5% (*n* = 21) were explicitly theory- or model-based. The three most popular models/frameworks were Plan-Do-Study-Act (PDSA), motivational interviewing (MI), and self-efficacy (SE). The PDSA model has been widely used in quality improvement projects; however, it is not a theoretical behavioral model by which an intervention should be developed, but rather a four-step, structured cyclical approach to test the outcome of a change that has been implemented [40,41]. The intended use of the PDSA model is small scale, where the cycle is rapidly repeated multiple times to test and refine a single element within a short duration and a small sample size [41,42]. Two separate systematic reviews of the model found that although widely used, many projects that use PDSA report improvements while not adhering to its methodology of four steps [41,43]. Additionally, the model itself is more rooted in healthcare processes than patient outcomes [41].

In contrast, MI is a behavior change counselling technique developed by clinical psychologists [44]. It is best described as a “person-centered, goal-oriented style of communication” with the goal of behavior change [45]. Originally derived to help those with alcoholism and addiction, robust data exist of MI working across various demographics and in the promotion of treatment adherence in chronic diseases [46]. With an emphasis on communication, MI requires direct patient contact, as it revolves around conversations between the patient and a trained healthcare provider [44].

Lastly, SE, developed by Bandura (1977), is an individual’s belief in their own ability to successfully perform a specific task related to a specific behavior (i.e., taking their medication on time to adhere to treatment) [47]. High self-efficacy is associated with a sense of mastery, accomplishment, and feeling in control [47]. Thus, an individual with a high level of self-efficacy toward a particular task is more optimistic about their ability to cope with unexpected events and challenges that may occur, whereas an individual with low self-efficacy may give up easily or avoid the task altogether. There exist both internal and external sources of self-efficacy, providing potential mechanisms for interventions [47]. A systematic review of the relationship between self-efficacy and medication adherence found a positive link between them in 59 out of 66 studies [48]. One publication included in this mapping review combined both SE and MI, as SE is a theory of human behavior, while MI is a technique. Interestingly, self-efficacy theory, with its significant positive associations with medication adherence [48], was mentioned in only three studies, while self-efficacy used as an outcome measure in six studies, suggesting that theory-driven work may not be explicitly identified in reporting.

We found a limited number of studies to be experimental, whereas the majority were quasi-experimental or observational. Prior to clinical implementation of OAA interventions, higher levels of evidence should be required. Studies should also consider mixed-method designs, as they may generate more comprehensive and complementary knowledge on intervention effects. Interestingly, nearly one-quarter of publications (23.3%) did not specify the types of OAAs used. Moving forward, researchers must be more explicit when reporting on OAAs so that replication studies and projects can be more easily carried out.

As expected, medication adherence was the most prevalent outcome used across studies. Methods to measure OAA adherence were primarily self-report or pharmacy refill rates. Self-report questionnaires are convenient and may allow adherence-related factors and behaviors to be considered. However, they may be subject to recall, response, and desirability biases that may overestimate adherence rates. Of the 30 studies that relied on self-report, only 11 (37%) used validated questionnaires. The use of study-specific questionnaires may be due in part to the large copyright and licensing fee researchers are asked to pay for the most commonly used validated self-report measure, the Morisky Medication Adherence Scale (MMAS-8) [49]. Originally developed in the context of anti-hypertensive drugs, the scale has been validated and widely used across various populations and languages [50]. The Medication Adherence Report Scale (MARS-5) and Medication Adherence Questionnaire (MAQ) are both free alternatives to the MMAS-8 [51,52]. Developed in 2023 by Talens et al., the Oral Chemotherapy Adherence Scale (OCAS) specifically assesses adherence to oral chemotherapy [53]. Validated in Spanish, the scale has yet to be validated in an English-speaking sample. If successfully validated, this scale may be the best option for OAA researchers. With questions such as “do you sometimes think that another intravenous drug would produce better results than the current drug?”, the scale captures elements specific to the OAA experience that generic adherence scales miss.

Pharmacy refill rates, while being objective, do not measure the uptake of medication itself. The two common refill measures, the medication possession ratio (MPR) and proportion of days covered (PDC), vary slightly, but with significant ramifications. The MPR—the sum of days’ supply for all fills in the period/number of days in the period—may overestimate adherence if a patient refills their medication a few days before the end of the previous period, thus making it possible to be higher than 100%. The PDC—the number of days covered/number of days in the period—adjusts the calculation and shifts overlapping days and is, therefore, the more accurate measurement [54]. However, of the 28 studies that relied on refill data, only 2 used PDC. To best mitigate risks associated with each indicator and obtain a more complete picture of adherence, a combined objective and subjective approach is ideal.

In the past two years, a systematic review [17], a systematic review and meta-analysis [18], as well as an overview of reviews (narrative synthesis) [16] were published to guide clinical practice on what interventions may improve medication adherence to OAAs. All three found the level of evidence to be too low, lacking in quality, as well as presenting a high risk of bias among studies. Our findings are indeed consistent with their recommendations for more rigorous, large-scale studies of theory-based interventions. As interventions continue to be developed and tested, this mapping review may serve to guide the first step of their process. The findings inform current gaps in the literature and the discussion provides context. Specific takeaways include self-efficacy as a potential intervention guiding model to be further explored, the four intervention modes (behavioral, educational, medical, and technological) to consider, linking the theory used to carefully selected outcome measures, and the variability that exists in the measurement of medication adherence.

### Limitations

Despite a rigorous methodology (PRISMA) and efforts to ensure transparency in coding, there are a few limitations to this review. All publications were identified, screened, and data were extracted by one author, a doctoral student, as part of their dissertation work. The defined coding framework for each category of data extracted did not go further into study details, such as sample characteristics (e.g., age, sex, or gender), as the purpose of this review was not comparative. Due to the scope of a mapping review, many publications were included with the main intervention components and testing described; however, specific study results were not included. There already exist many systematic reviews and meta-analyses on the topic, focusing exclusively on results. This mapping review is not meant to guide clinical practice in terms of what interventions may work best, but rather guide future work in the field. Lastly, the publications stemmed from four well-established databases with large repositories, though it is possible that studies from other databases were missed.

## 6. Conclusions

As cancer care moves increasingly toward precision medicine, more anticancer agents will be taken orally. As such, we are likely to see more OAA studies with new drugs. It is critical that research on this topic be thoroughly conducted, favoring theory-driven experimental and mixed-method approaches, with careful consideration to outcome selection. The rigorous accumulating evidence will help determine if (and which) supportive interventions significantly optimize OAA adherence and health-related outcomes for patients.

## Figures and Tables

**Table 1:** PICO search strategy guiding the mapping review [23].

Concept 1 Patient/Population	Concept 2 Intervention/Exposure	Concept 3 Outcome
Oral anticancer agent	Supportive intervention	Medication adherence

**Table 2:** Theories and frameworks used in study and/or intervention development of the included publications (N = 120).

Theory/Framework	Number of Publications N = 120, *n* (%)
Plan-Do-Study-Act (PDSA) Model	4 (3.3)
Self-efficacy	3 (2.5)
Motivational interviewing	2 (1.6)
Self-efficacy and motivational interviewing	1 (0.83)
Acceptance and Commitment Therapy (ACT) and self-affirmation theory	1 (0.83)
Concordance and shared decision-making	1 (0.83)
Conceptual framework created to study med adherence	1 (0.83)
Health belief model and the stress process model	1 (0.83)
Intervention for Symptom Management Model	1 (0.83)
Motivation Theory	1 (0.83)
Self-Care-Deficient Nursing Theory	1 (0.83)
Self-Regulatory Model of Antiretroviral Adherence	1 (0.83)
Social Representation (SR) theory	1 (0.83)
Synergy Model of Patient Care and Ottawa Model of Research Use	1 (0.83)
UK Medical Research Council’s Self-Management Framework	1 (0.83)

**Table 3:** Summary of study designs (N = 120).

Study Design	Number of Publications, N = 120, *n* (%)
Observational, cohort	47 (39.2)
Quasi-experimental	46 (38.3)
Experimental	20 (16.7)
Qualitative	4 (3.3)
Observational, cross-sectional	3 (2.5)

**Table 4:** Summary of OAA types (N = 120).

OAA Drug Types	Number of Publications, N = 120, *n* (%)
Systemic and targeted	33 (27.5)
Drugs not specified	28 (23.3)
Targeted	17 (14.2)
Capecitabine/Xeloda only	13 (10.8)
Systemic, targeted, and hormonal	3 (2.5)
Hormonal	5 (4.2)
Combination treatment of IV and PO	3 (2.5)

**Table 5:** Summary of intervention modalities (N = 120).

Intervention Modality	Number of Publications, N = 120, *n* (%)
Pharmacist-led	41 (34.2)
Nurse-led	22 (18.3)
Digital health	25 (20.8)
Multi-disciplinary	20 (16.6)
Digital health/healthcare provider combined	8 (6.7)
Paper-based	4 (3.3)

**Table 6:** List of measures, including self-report, chart refill, and combined methods of adherence.

Self-Report (*n* = 31)	Refill/Chart (*n* = 28)	Combination (*n* = 7)
Study-specific questionnaire (*n* = 16)	Medication possession ratio: MPR (*n* = 16)	Diary + pill count (*n* = 1)
Morisky Medication Adherence Scale: MMAS-8 (*n* = 7)	Details not available: NA (*n* = 7)	Self-report (Basel Assessment of Adherence Scale: BAAS) + pill count (*n* = 1)
Via telephone (*n* = 2)	Proportion of days covered: PDC (*n* = 2)	Self-report (MARS-5) + e-pillbox (*n* = 1)
Medication Adherence Report Scale: MARS-5 (*n* = 1)	Relative dose intensity: RDI (*n* = 2),	Self-report (MMAS-8) + e-pillbox (*n* = 1)
Oral Chemotherapy Adherence Scale: OCAS (*n* = 1)	MPR/PDC/TTT (*n* = 1)	Self-report (study-specific) + refill (NA) (*n* = 1)
Medication Adherence Questionnaire: MAQ (*n* = 1)		Plasma drug concentration + self-report (study-specific) (*n* = 1)
Morisky Green Levine Medication Adherence Scale (*n* = 1)		RDI + pill count (*n* = 1)
Patient diary (*n* = 1)		

## Supplementary Materials

The following are available online at https://www.mdpi.com/article/10.3390/curroncol30120744/s1, File S1. Publications included in the mapping review (N = 120): year author(s), outcome(s) of interest, and adherence measure.
