# Supplementary material for: Patient Adherence to Oral Anticancer Agents: A Mapping Review of Supportive Interventions
Source: Curr Oncol. 2023 Nov 30;30(12):10224–36. doi: 10.3390/curroncol30120744 (PMC10743037; doi:10.3390/curroncol30120744)
Supplement: Supplementary file 1 [file curroncol-30-00744-s001.zip › curroncol-2697831-supplementary.pdf]

Supplementary Material

Publications included in the mapping review (N = 120): year, reference, outcome(s) of interest, and adherence measure.

Year	Reference	Outcome(s) of Interest
2022	Accordino M.K., Ulene S., Honan E., Trivedi M.S., Crew K.D., Harden E., Law, C., Hershman, D.L. (2022). Feasibility and patient satisfaction with a smartphone application to improve medication adherence among patients with breast cancer. Cancer Research, 82(4 SUPPL), no pagination. https://doi.org/10.1158/1538-7445.SABCS21-P4-11-16	• Feasibility: Intervention completion rates<br>• Patient satisfaction: User Mobile Application Rating Scale (uMARS)
2022	Arch, J. J., Crespi, C. M., Levin, M. E., Genung, S. R., Nealis, M., Mitchell, J. L., Bright, E. E., Albright, K., Magidson, J. F., & Stanton, A. L. (2022). Randomized Controlled Pilot Trial of a Low-Touch Remotely-Delivered Values Intervention to Promote Adherence to Adjuvant Endocrine Therapy Among Breast Cancer Survivors. Annals of behavioral medicine : a publication of the Society of Behavioral Medicine, 56(8), 856–871. https://doi-org.proxy3.library.mcgill.ca/10.1093/abm/kaab118	• Adherence: Smart pillbox
2022	Billings P., Shaunak N., Oakley C., Coughlan C. & Shah T. (2022). Oral Systemic Anti-Cancer Therapy (SACT) clinic to enhance patients' pathway for cancer patients. Journal of Oncology Pharmacy Practice, 28(2 SUPPL), 51-52. https://doi.org/10.1177/10781552221078082	• Adherence: Self-report via telephone call<br>• Confidence in taking medicine and managing/recognizing side effects<br>• Oncology Services awareness/support<br>• Patients' satisfaction
2022	Bright, E. E., Genung, S. R., Stanton, A. L., & Arch, J. J. (2022). A mixed-methods study of the technical feasibility and patient acceptability of a real-time adherence monitor in breast cancer survivors taking adjuvant endocrine therapy. Breast cancer research and treatment, 195(3), 393–399. https://doi-org.proxy3.library.mcgill.ca/10.1007/s10549-022-06705-1	• Adherence: Smart pillbox
2022	Dennis, M., Haines, A., Johnson, M., Soggee, J., Tong, S., Parsons, R., Sunderland, B., & Czarniak, P. (2022). Cross-sectional Census Survey of Patients With Cancer who Received a Pharmacist Consultation in a Pharmacist Led Anti-cancer Clinic. Journal of cancer education : the official journal of the American Association for Cancer Education, 37(5), 1553–1561. https://doi-org.proxy3.library.mcgill.ca/10.1007/s13187-022-02196-2	• Patient perceptions and satisfaction<br>• Adherence: Self-report via questionnaire
2022	El-Behadli, Ana F, Germann, Julie N, Pratt, Chelsea, Acosta, Dailyn, Montiel-Esparza, Raul, Alvarez, Nancy, et al. (2022). Culturally adapted motivational interviewing for pediatric acute lymphoblastic leukemia adherence: Feasibility and acceptability. Clinical Practice in Pediatric Psychology, No Pagination Specified. https://doi.org/10.1037/cpp0000447	• Feasibility and acceptability<br>• Adherence: Self-report via questionnaire
2022	Feral, A., Boone, M., Lucas, V., Bihan, C., Belhout, M., Chauffert, B., & Lenglet, A. (2022). Influence of the implementation of a multidisciplinary consultation program on adherence to the first ever course of oral antineoplastic treatment in patients with cancer. Journal of oncology pharmacy practice : official publication of the International Society of Oncology Pharmacy Practitioners, 28(7), 1543–1551. https://doi-org.proxy3.library.mcgill.ca/10.1177/10781552211035368	• Adherence: Medication possession ratio (MPR)<br>• Adverse events

2022	Keating, N. L., Brooks, G. A., Landrum, M. B., Liu, P. H., Wolf, R., Riedel, L. E., Kapadia, N. S., Jhatakia, S., Tripp, A., Simon, C., Hsu, V. D., Kummet, C. M., & Hassol, A. (2022). The Oncology Care Model and Adherence to Oral Cancer Drugs: A Difference-in-Differences Analysis. Journal of the National Cancer Institute, 114(6), 871–877. https://doi-org.proxy3.library.mcgill.ca/10.1093/jnci/djac026	• Adherence: Proportion of days covered (PDC) via Medicare data
2022	Lau-Min K.S., Marini J., Shah N., Pucci D., Blauch A., Cambareri C., et al (2022). An augmented intelligence mobile phone chatbot for medication adherence and toxicity management among patients with gastrointestinal cancers on capecitabine. Journal of Clinical Oncology, 40(28 Supplement), 424. https://doi.org/10.1200/JCO.2022.40.28_suppl.424	Primary:<br>• Feasibility: Completion of cohort without safety events<br>Secondary:<br>• Adherence: Self-report via questionnaire<br>• Engagement
2022	Lichtenstein M.R.L., Patel K., Campbell P., Nguyen M.K., Harden E., Spivack J., et al (2022). Evaluation of a pharmacist-led video consultation to identify drug interactions among patients initiating oral anticancer drugs. Journal of Clinical Oncology, 40(16 Supplement 1), no pagination. https://doi.org/10.1200/JCO.2022.40.16_suppl.1592	• Drug–drug interactions<br>• Polypharmacy<br>• Patient satisfaction
2022	Lory, P., Perche, L., Blanc, J., Fouquier, B., Giroux, A., Thomassin, A., Devaux, M., Renaudin, A., Di Martino, C., Quipourt, V., Bengrine-Lefèvre, L., & Schmitt, A. (2022). Adherence to oral anti-cancer therapies in older patients is similar to that of younger patients. Journal of oncology pharmacy practice : official publication of the International Society of Oncology Pharmacy Practitioners, 10781552221103547. Advance online publication. https://doi-org.proxy3.library.mcgill.ca/10.1177/10781552221103547	• Medication adherence: Patient diary<br>• Medication adherence: Pill count
2022	McAuliff K., Rutter W., Cavers W., Shah D., Pittos E., Feczko L., et al (2022). Impact of the COVID-19 pandemic on oral oncolytic adherence. Journal of Clinical Oncology, 40(28 Supplement), 395. https://doi.org/10.1200/JCO.2022.40.28_suppl.395	Primary:<br>• Adherence: MPR<br>Secondary:<br>• Digital engagement: Number of times interacted with platform
2022	Mathur, A. D., Maiers, T. A., & Andrick, B. J. (2022). Impact of a pharmacist-led telehealth oral chemotherapy clinic. American journal of health-system pharmacy : AJHP : official journal of the American Society of Health-System Pharmacists, 79(11), 896–903. https://doi.org/10.1093/ajhp/zxac038	Number of:<br>• Patients seen<br>• Referrals made<br>• Times education sessions were conducted
2022	Park, D., Patel, S., Yum, K., Smith, C. B., Tsao, C. K., & Kim, S. (2022). Impact of Pharmacist-Led Patient Education in an Ambulatory Cancer Center: A Pilot Quality Improvement Project. Journal of pharmacy practice, 35(2), 268–273. https://doi-org.proxy3.library.mcgill.ca/10.1177/0897190020970770	• Anxiety<br>• Patient understanding and knowledge
2022	Patel, J. V., Hughes, D. M., & Ko, N. Y. (2022). OPTIMAL Breast Cancer Care: Effect of an Outpatient Pharmacy Team to Improve Management and Adherence to Oral Cancer Treatment. JCO oncology practice, OP2200135. Advance online publication. https://doi-org.proxy3.library.mcgill.ca/10.1200/OP.22.00135	• Adherence to lab parameter monitoring<br>• Number of interventions per patient<br>• Overall time on therapy
2022	Porcher, L., Perron, V., Blanc, J., Kaderbhai, C. G., Tharin, Z., Schmitt, A., & Gallet, M. (2022). Smartphone-based application and nurses' interventions for symptoms monitoring in patients treated with oral anticancer agents: A 1-year follow-up in a tertiary cancer center. Journal of oncology pharmacy practice : official publication of the International Society of Oncology Pharmacy Practitioners, 10781552221117731. Advance online publication. https://doi-org.proxy3.library.mcgill.ca/10.1177/10781552221117731	• Adherence: Self-report via questionnaire<br>• Alerts and nursing intervention type<br>• Symptoms: Grade and type

2022	Sargent, W., & Whalley, A. (2022). Implementation and outcomes of a pharmacist-led oral chemotherapy clinic at VA Maine Healthcare System. Journal of oncology pharmacy practice : official publication of the International Society of Oncology Pharmacy Practitioners, 28(8), 1704–1708. https://doi-org.proxy3.library.mcgill.ca/10.1177/10781552211039501	• Adherence: MPR<br>• Intervention type
2022	Signorelli, J., Bell, C., & Monaco, S. (2022). Oral oncolytic monitoring pilot with patient-reported outcomes and adherence assessments. Journal of oncology pharmacy practice : official publication of the International Society of Oncology Pharmacy Practitioners, 10781552221112603. Advance online publication. https://doi-org.proxy3.library.mcgill.ca/10.1177/10781552221112603	• Adherence: Self-report via MMAS-8<br>• PROs: ESAS-r
2022	Skrabal Ross, X., Gunn, K. M., Suppiah, V., Patterson, P., Boyle, T., Carrington, C., Tan, S. L., Ryan, M., Joshi, R., & Olver, I. (2022). A smartphone program to support adherence to oral chemotherapy in people with cancer: Proof-of-concept trial. Asia-Pacific journal of clinical oncology, 18(5), e378–e387. https://doi-org.proxy3.library.mcgill.ca/10.1111/ajco.13656	• Acceptability and satisfaction with the intervention<br>• Adherence: Self-report via MARS-5<br>• Adherence: Electronic smart pillbox<br>• Knowledge<br>• Side effects' presence and severity
2022	Zhang, Y., Zou, W., Wu, X., Wang, X., Zhang, M., Wu, X., Qin, H., & Zhang, M. (2022). Effect of hospital-based case management on psychosocial wellbeing and treatment outcomes in colorectal cancer patients: A quasi-experimental study. International journal of nursing practice, 28(6), e13104. https://doi-org.proxy3.library.mcgill.ca/10.1111/ijn.13104	• Adherence: Self-reported via the Morisky Adherence Questionnaire (MAQ)<br>• Anxiety and depression<br>• Quality of life<br>• Symptom distress: MD-ADI<br>• Unplanned readmission
2021	Bana M. & Szuts N. (2021). Evaluation and experience with nurse-led follow-up phone calls for oral tumor therapies. Oncology Research and Treatment, 44(SUPPL 2), 306. https://doi.org/10.1159/000518417	• Number of nurse consults and phone calls
2021	Bouleftour, W., Muron, T., Guillot, A., Tinquaut, F., Rivoirard, R., Jacquin, J. P., Saban-Roche, L., Boussoualim, K., Tavernier, E., Augeul-Meunier, K., Collard, O., Mery, B., Pupier, S., Oriol, M., Bourmaud, A., Fournel, P., & Vassal, C. (2021). Effectiveness of a nurse-led telephone follow-up in the therapeutic management of patients receiving oral antineoplastic agents: a randomized, multicenter controlled trial (ETICCO study). Supportive care in cancer : official journal of the Multinational Association of Supportive Care in Cancer, 29(8), 4257–4267. https://doi-org.proxy3.library.mcgill.ca/10.1007/s00520-020-05955-3	• Adherence: Self-report via the Morisky Medication Adherence Scale (MMAS-8)<br>• Quality of Life (QoL): Eq-5D<br>• Toxicity
2021	Brice, K.Y. (2021) Development and Evaluation of a Nurse Practioner-Directed Telephone Follow-Up Initiative to Improve Oral Chemotherapy Compliance in an Outpatient Oncology Practice. [Doctoral dissertation, Wilmington University]. ProQuest Dissertations Publishing.	• Adherence: Chart review
2021	Doolin, J. W., Berry, J. L., Forbath, N. S., Tocci, N. X., Dechen, T., Li, S., Hartwell, R. A., Espiritu, J. K., Roberts, D. A., Zerillo, J. A., & Shea, M. (2021). Implementing Electronic Patient-Reported Outcomes for Patients With New Oral Chemotherapy Prescriptions at an Academic Site and a Community Site. JCO clinical cancer informatics, 5, 631–640. https://doi-org.proxy3.library.mcgill.ca/10.1200/CCI.20.00191	• Time to first symptom assessment
2021	Gallagher, Emily Elizabeth. (2021). Oral chemotherapy patient education using the multinational association of supportive care in cancer oral agent teaching tool. Dissertation Abstracts International: Section B: The Sciences and Engineering, 82(12-B) , No Pagination Specified. Retrieved from http://ovidsp.ovid.com/ovidweb.cgi?T=JS&PAGE=reference&D=psyc18&NEWS=N&AN=2021-65615-039.	• Adherence: Self-report via Adherence Starts with Knowledge scale (ASK-12)

2021	Ghiggia, A., Bianco, A., Castelli, L., Baratta, F., Birocco, N., Scaldaferri, M., Milla, P., Tesio, V., Rosso, S., Torta, R., Brusa, P., & Cattel, F. (2021). Adherence to oral chemotherapy: Evidence from a randomised clinical trial. European journal of cancer care, 30(1), e13336. https://doi-org.proxy3.library.mcgill.ca/10.1111/ecc.13336	• Adherence: Plasma drug concentration<br>• Adherence: Self-report via questionnaire<br>• Psychological assessment: Coping strategies, distress, personality traits
2021	Gönderen Çakmak, H. S., & Kapucu, S. (2021). The Effect of Educational Follow-Up with the Motivational Interview Technique on Self-Efficacy and Drug Adherence in Cancer Patients Using Oral Chemotherapy Treatment: A Randomized Controlled Trial. Seminars in oncology nursing, 37(2), 151140. https://doi-org.proxy3.library.mcgill.ca/10.1016/j.soncn.2021.151140	• Adherence: Self-report via Oral Chemotherapy Adherence Scale (OCAS)<br>• Medication adherence self-efficacy: Medication Adherence Self-Efficacy Scale (MASES)
2021	Healy R., Passey D., Pinnell D., Qualls J., Hamilton C., Burningham Z., et al (2021). Veterans on anticancer medications in rural and community environment support (VA CARES) program: A pharmacist-led telemedicine medication management program for veterans receiving oral antineoplastic therapies through the MISSION Act. Journal of Clinical Oncology, 39(15 SUPPL), no pagination. https://doi.org/10.1200/JCO.2021.39.15_suppl.1545	• Economic benefits: Cost savings or cost avoidance<br>• Patient satisfaction<br>• Safety: Number and type of pharmacist interventions
2021	Johengen E., Davidson A., Beekman K.W., Hecht K. & MacKler E.R. (2021). Improvement in time to oral anticancer agent follow-up. Journal of Clinical Oncology, 39(28 SUPPL), no pagination. https://doi.org/10.1200/JCO.2020.39.28_suppl.235	Time to follow-up
2021	Karaaslan-Eşer, A., & Ayaz-Alkaya, S. (2021). The effect of a mobile application on treatment adherence and symptom management in patients using oral anticancer agents: A randomized controlled trial. European journal of oncology nursing : the official journal of European Oncology Nursing Society, 52, 101969. https://doi-org.proxy3.library.mcgill.ca/10.1016/j.ejon.2021.101969	• Adherence: Self-report via Oral Chemotherapy Adherence Scale (OCAS),<br>• Symptoms: Memorial Symptom Assessment Scale (MSAS)
2021	Kongshaug, N., Skolbekken, J. A., Faxvaag, A., & Hofsli, E. (2021). Cancer Patients' Perceived Value of a Smartphone App to Enhance the Safety of Home-Based Chemotherapy: Feasibility Study. JMIR formative research, 5(1), e20636. https://doi-org.proxy3.library.mcgill.ca/10.2196/20636	• Patients perceptions: Focus groups/interviews (qualitative)
2021	Lin, M., Hackenyos, D., Savidge, N., Weidner, R. A., Murphy-Banks, R., Fleckner, T., Parsons, S. K., & Rodday, A. M. (2021). Enhancing patients' understanding of and adherence to oral anticancer medication: Results of a longitudinal pilot intervention. Journal of oncology pharmacy practice : official publication of the International Society of Oncology Pharmacy Practitioners, 27(6), 1409–1421. https://doi-org.proxy3.library.mcgill.ca/10.1177/1078155220960800	• Adherence: Self-report via questionnaire<br>• Understanding
2021	Nhean, S., Kostoff, D., Yang, J. J., Vogel, V., & Rybkin, I. I. (2021). Impact of Oral Chemotherapy Management Program on Capecitabine Toxicity Management. JCO oncology practice, 17(7), e1021–e1029. https://doi-org.proxy3.library.mcgill.ca/10.1200/OP.20.00947	Primary:<br>• Adverse events<br>Secondary:<br>• Adherence: medication possession ratio (MPR)<br>• ER visits/hospitalizations due to toxicity
2021	Psihogios, A. M., Li, Y., Ahmed, A., Huang, J., Kersun, L. S., Schwartz, L. A., & Barakat, L. P. (2021). Daily text message assessments of 6-mercaptopurine adherence and its proximal contexts in adolescents and young adults with leukemia: A pilot study. Pediatric blood & cancer, 68(2), e28767. https://doi-org.proxy3.library.mcgill.ca/10.1002/pbc.28767	• Adherence: Smart pillbox<br>• Feasibility and acceptability: Recruitment/retention rates, cost, and technical issues

2021	Rasschaert, M., Vulsteke, C., De Keersmaeker, S., Vandenborne, K., Dias, S., Verschaeve, V., Vuylsteke, P., Brussel, I. V., Ravelingien, J., Dam, P. V., Segelov, E., & Peeters, M. (2021). AMTRA: a multicentered experience of a web-based monitoring and tailored toxicity management system for cancer patients. Supportive care in cancer : official journal of the Multinational Association of Supportive Care in Cancer, 29(2), 859–867. https://doi-org.proxy3.library.mcgill.ca/10.1007/s00520-020-05550-6	• Compliance: Self-report via app<br>• Toxicity
2021	Rubira, L., Leenhardt, F., Perrier, C., & Pinguet, F. (2021). Sécurisation du parcours de soins du patient sous thérapie orale en oncologie : expérimentation autour d'un lien pharmaceutique hôpital–ville [Securing the patient's care path receiving oral anticancer therapy: Experimentation around a pharmaceutical hospital-to-community liaison]. Annales pharmaceutiques francaises, 79(5), 558–565. https://doi-org.proxy3.library.mcgill.ca/10.1016/j.pharma.2021.01.009	• Use of tools over one year: Number of pharmacists contacted and follow-ups
2021	Sun, W., Reeve, R., Ouellette, T., Stutsky, M., De Jesus, R., Huffer, M. J., & Mougalian, S. S. (2021). Novel Tool to Monitor Adherence to Oral Oncolytics: A Pilot Study. JCO clinical cancer informatics, 5, 701–708. https://doi-org.proxy3.library.mcgill.ca/10.1200/CCI.20.00151	• Adherence: Self-report via questionnaire
2020	Bowles, H., Tawfik, B., Abernathy, J., Lauer, R., Hashemi, N., & Dayao, Z. (2020). Pharmacist-Driven Oral Oncolytic Medication Education and Consent. JCO oncology practice, 16(10), e1209–e1215. https://doi-org.proxy3.library.mcgill.ca/10.1200/JOP.19.00418	• Education<br>• Consent rate
2020	Chouinard, A., Charpentier, D., Doucet, S., Messier, C., & Vachon, M. F. (2020). From theory to practice: implementing a standardized, interactive education session on oral anticancer medication (OAM) for patients and their caregivers. Supportive care in cancer : official journal of the Multinational Association of Supportive Care in Cancer, 28(8), 3897–3904. https://doi-org.proxy3.library.mcgill.ca/10.1007/s00520-019-05236-8	• Patients' level of confidence related to regimen
2020	Collado-Borrell, R., Escudero-Vilaplana, V., Ribed, A., Gonzalez-Anleo, C., Martin-Conde, M., Romero-Jimenez, R., Iglesias-Peinado, I., Herranz-Alonso, A., & Sanjurjo-Saez, M. (2020). Effect of a Mobile App for the Pharmacotherapeutic Follow-Up of Patients With Cancer on Their Health Outcomes: Quasi-Experimental Study. JMIR mHealth and uHealth, 8(10), e20480. https://doi-org.proxy3.library.mcgill.ca/10.2196/20480	• Adherence: Medication possession ratio (MPR)<br>• Drug-related problems and side effects<br>• Quality of life: EQ-5D<br>• Side effects
2020	Curry, M. A., Chineke, I., Redelico, T., Terrell, C., Bell, W., Flood, D., Mishra, P., LaFollette, J., Power, S., & Bernal-Mizrachi, L. (2020). Adherence to Oral Anticancer Medications After Implementation of an Ambulatory Adherence Program at a Large Urban Academic Hospital. JCO oncology practice, 16(4), e350–e356. https://doi-org.proxy3.library.mcgill.ca/10.1200/JOP.19.00167	• Adherence: Medication possession ratio (MPR), ER visits, and hospitalization<br>• Pharmacist interventions
2020	Deluche, E., Darbas, T., Bourcier, K., Montangon, L., Bayard, G., Caille, E., Querrioux, J., Suchaud, C., Zabaleta, S., Chaput, S., Le Brun-Ly, V., Pestre, J., Venat, L., Thuillier, F., Nevado, E., Maillan, G., Jost, J., Leobon, S., Tubiana-Mathieu, N., & Lavau-Denes, S. (2020). Prospective evaluation of an anti-cancer drugs management programme in a dedicated oral therapy center (DICTO programme). Medical oncology (Northwood, London, England), 37(8), 69. https://doi-org.proxy3.library.mcgill.ca/10.1007/s12032-020-01393-7	• Nurse consults: Time to consult and follow-up care

2020	Greer, J. A., Jacobs, J. M., Pensak, N., Nisotel, L. E., Fishbein, J. N., MacDonald, J. J., Ream, M. E., Walsh, E. A., Buzaglo, J., Muzikansky, A., Lennes, I. T., Safren, S. A., Pirl, W. F., & Temel, J. S. (2020). Randomized Trial of a Smartphone Mobile App to Improve Symptoms and Adherence to Oral Therapy for Cancer. Journal of the National Comprehensive Cancer Network : JNCCN, 18(2), 133–141. https://doi-org.proxy3.library.mcgill.ca/10.6004/jnccn.2019.7354	• Adherence: Electronic pill caps<br>• QoL: FACT-G<br>• Symptoms: MDSI
2020	Junior M.M., Pegnolato S., Lodi L.A., Marcolino M. & Fonseca R.P. (2020). Pharmacist assistance for patients on oral oncologic therapy: Impact on adherence and costs. Journal of Clinical Oncology, 38(15), no pagination. https://doi.org/10.1200/JCO.2020.38.15_suppl.e19198	• Adherence: PDC
2020	Komatsu, H., Yagasaki, K., Yamaguchi, T., Mori, A., Kawano, H., Minamoto, N., Honma, O., & Tamura, K. (2020). Effects of a nurse-led medication self-management programme in women with oral treatments for metastatic breast cancer: A mixed-method randomised controlled trial. European journal of oncology nursing : the official journal of European Oncology Nursing Society, 47, 101780. https://doi-org.proxy3.library.mcgill.ca/10.1016/j.ejon.2020.101780	Primary:<br>• Adherence: MPR<br>Secondary:<br>• HRQoL: fact-b<br>• Self-efficacy: General self-efficacy scale<br>• Symptoms: Mdasi
2020	McCabe, C. C., Barbee, M. S., Watson, M. L., Billmeyer, A., Lee, C. E., Rupji, M., Chen, Z., Haumschild, R., & El-Rayes, B. (2020). Comparison of rates of adherence to oral chemotherapy medications filled through an internal health-system specialty pharmacy vs external specialty pharmacies. American journal of health-system pharmacy : AJHP : official journal of the American Society of Health-System Pharmacists, 77(14), 1118–1127. https://doi-org.proxy3.library.mcgill.ca/10.1093/ajhp/zxaa135	• Adherence: MPR<br>• Adherence: PDC<br>• Adherence: TTT
2020	Marmorat, T., Rioufol, C., Ranchon, F., & Préau, M. (2020). Encounters between medical and lay knowledge in therapeutic patient education. A qualitative study based on an oral chemotherapy program. Patient education and counseling, 103(3), 537–543. https://doi-org.proxy3.library.mcgill.ca/10.1016/j.pec.2019.10.012	• Knowledge sharing<br>• Self-care and psychosocial skills
2020	Menhorn T., Zarick-Jones A., Turquie M.H., Sasankan S., Hashemi-Sadraei N., Dayao Z.R., Lauer, R.C., Tawfik, B., Bowles, H., Crozier, N. (2020). Oral oncolytic education and adherence monitoring in poor, rural and minority patients. Journal of Clinical Oncology, 38(15), no pagination. https://doi.org/10.1200/JCO.2020.38.15_suppl.e19212	• Timing of education
2020	Mir O., Ferrua M., Fourcade A., Mathivon D., Duflot-Boukobza A., Dumont S.N., et al (2020). Intervention combining nurse navigators (NNs) and a mobile application versus standard of care (SOC) in cancer patients (pts) treated with oral anticancer agents (OAA): Results of CapRI, a single-center, randomized phase III trial. Journal of Clinical Oncology, 38(15), no pagination. https://doi.org/10.1200/JCO.2020.38.15_suppl.2000	Primary:<br>• Adherence: Relative dose intensity (RDI)<br>Secondary:<br>• Toxicity<br>• Response and survival<br>• Quality of life<br>• Pts experience (PACIC Score)<br>• End-of-life support<br>• Economic estimation of the use of healthcare resources
2020	Sohal M., McLarty S., Friend K.E., Johnson K.D., Johlie M., Sawicki C., et al (2020). Using digital engagement to proactively manage symptoms in patients on capecitabine. Journal of Clinical Oncology, 38(15), no pagination. https://doi.org/10.1200/JCO.2020.38.15-suppl.12079	• Adherence: PDC

2019	Babin, M., Folliard, C., Robert, J., Sorrieul, J., Kieffer, H., Augereau, P., & Devys, C. (2019). Consultations pharmaceutiques en oncologie : mise en place, bilan à un an et perspectives [Pharmaceutical consultations in oncology: Implementation, one-year review and outlooks]. Annales pharmaceutiques francaises, 77(5), 426–434. https://doi-org.proxy3.library.mcgill.ca/10.1016/j.pharma.2019.05.001	• Number of consults and number of pharmaceutical interventions
2019	Baron J., Lombardi C.L., Yu H., Przespolewski A., Griffiths E.A., Thompson J.E., et al (2019). Benefits of a Pharmacist Led Oral Chemotherapy Monitoring Program for Patients with Chronic Myeloid Malignancies: A Patient Reported Outcome (PRO) Study. Blood, 134(Supplement 1), 3501. https://doi.org/10.1182/blood-2019-131629	• HRQoL: FACT-Leu<br>• Perceptions/usefulness of program
2019	Conliffe, B., Figg, L., Moffett, P., Lauterwasser, L., & Parsons, L. B. (2019). Impact of a formal pharmacist-run oral antineoplastic monitoring program: A pilot study in an adult genitourinary oncology clinic. Journal of oncology pharmacy practice : official publication of the International Society of Oncology Pharmacy Practitioners, 25(4), 777–786. https://doi-org.proxy3.library.mcgill.ca/10.1177/1078155217753889	• Adherence/persistence: Chart review<br>• Meeting recommendations: Chart review<br>• Seeking medical care and interventions: Chart review
2019	Corbett M., Arcieri C., Dann E., Durney J., Fuller F., Leonard F., et al (2019). Oral anti-cancer therapy initiation: A standardized approach for patient care and education. JNCCN Journal of the National Comprehensive Cancer Network, 17(3-5), no pagination. https://doi.org/10.6004/jnccn.2018.7113	• Acceptability/satisfaction
2019	Durr P., Schlichtig K., Dorje F. & Fromm M.F. (2019). Medication safety in patients treated with new oral antitumor agents: A prospective, randomized investigation on the impact of intensified clinical pharmaceutical/clinical pharmacological care on patient safety and well-being (AMBORA-Study-supported by the German Cancer Aid (70112447)). Naunyn-Schmiedeberg's Archives of Pharmacology, 392(Supplement 1), S14. https://doi.org/10.1007/s00210-01-0121-6	• Number of drug-related problems<br>• Patient satisfaction
2019	Eldeib, H. K., Abbassi, M. M., Hussein, M. M., Salem, S. E., & Sabry, N. A. (2019). The Effect of Telephone-Based Follow-Up on Adherence, Efficacy, and Toxicity of Oral Capecitabine-Based Chemotherapy. Telemedicine journal and e-health : the official journal of the American Telemedicine Association, 25(6), 462–470. https://doi-org.proxy3.library.mcgill.ca/10.1089/tmj.2018.0077	• Adherence: Pill count<br>• Toxicity: CTCAE
2019	Gerardin E., Protesti E., Cohen-Valensi R., Martinez S. & Berod T. (2019). Chemotherapy pharmaceutical consultation: Pharmaceutical interventions after 18 months of implementation. European Journal of Hospital Pharmacy, 26(Supplement 1), A140. https://doi.org/10.1136/ejhpharm-2019-eahpconf.301	• Medication-related problems<br>• Pharmaceutical consultations
2019	Hartwell R., Cibotti C., Roberts D., Doolin J., Yenulevich M., Trillo K., et al (2019). Impact of pharmacist-led oral antineoplastic patient education on compliance with quality oncology practice initiative (QOPI) measures. Journal of Oncology Pharmacy Practice, 25(3 Supplement), 16. https://doi.org/10.1177/1078155218823168	• Compliance with ACSO standards<br>• Number of interventions

2019	Linder, L. A., Wu, Y. P., Macpherson, C. F., Fowler, B., Wilson, A., Jo, Y., Jung, S. H., Parsons, B., & Johnson, R. (2019). Oral Medication Adherence Among Adolescents and Young Adults with Cancer Before and Following Use of a Smartphone-Based Medication Reminder App. Journal of adolescent and young adult oncology, 8(2), 122–130. https://doi-org.proxy3.library.mcgill.ca/10.1089/jayao.2018.0072	• Adherence: Patterns via electronic pill caps
2019	Maritaz, C., Gault, N., Roy, C., Tubach, F., Burnel, S., Lotz, J. P., & CHIMORAL (2019). Impact d'une organisation régionale coordonnée pour sécuriser la prise en charge des patients sous anticancéreux oraux : CHIMORAL, une étude comparative [Impact of a coordinated regional organization to secure the management of patients on oral anticancer drugs: CHIMORAL, a comparative trial]. Bulletin du cancer, 106(9), 734–746. https://doi-org.proxy3.library.mcgill.ca/10.1016/j.bulcan.2019.03.019	• Use of hospital for adverse events within 6 months of treatment
2019	McLarty S., Friend K.E., Chadha N., Verma S.K., Sawicki C. & Johnson K.A. (2019). Effect of secure clinical messaging on chemotherapy adherence. Journal of Clinical Oncology, 37(Supplement 15), no pagination. https://doi.org/10.1200/JCO.2019.37.15_suppl.e18542	• Adherence: MPR
2019	Montiel-Esparza R., El-Behadli A., Pratt C., Alvarez N., Saenz M.G., Germann J., et al (2019). Improving adherence to oral chemotherapy in all with motivational interviewing: A feasibility pilot. Pediatric Blood and Cancer, 66(Supplement 2), S188-S189. https://doi.org/10.1002/pbc.2771	• Knowledge<br>• Patient ratings
2019	Moreira G., Ferreira C.G., Montella T., Vasconcellos J. & Goncalves I. (2019). P1.26 Pharmaceutical Follow-up Program for Patients with Oral Drug Treatment in Non-small Cell Lung Cancer in a Heterogeneous Health Care System. Journal of Thoracic Oncology, 14(11 Supplement 2), S1184. https://doi.org/10.1016/j.jtho.2019.09.161	• Hospitalizations<br>• Toxicities
2019	Zukin M., Simoes Travassos Soares M.C., Gamboa N.F., Pombo F.H. & Carvalho L. (2019). Oral chemotherapy follow up on a Brazilian private health care unit. Journal of Clinical Oncology, 37(Supplement 15), no pagination. https://doi.org/10.1200/JCO.2019.37.15_suppl.e18311	• Adherence: Self-report via the Morisky Medication Adherence Scale (MMAS-8)
2018	Do L., Hiroo K. & White J. (2018). A pharmacist-monitored oral chemotherapy program at a community hospital. JACCP Journal of the American College of Clinical Pharmacy, 1(2), 314-315. https://doi.org/10.1002/jac5.1059	Acceptability<br>• Patients and oncologist
2018	Duffy A. & Gilmore S.A. (2018). Evaluating the inpatient of an oral chemotherapy standardized process improvement tool on patient-perceived ability to adhere to oral chemotherapy treatment plans. Journal of Oncology Pharmacy Practice, 24(2 Supplement 1), 6-7. https://doi.org/10.1177/1078155217751308	Patient perspective:<br>• Patient perceived ability to adhere to and handle medication safely (not explicitly stated: self-efficacy)<br>• Barriers/facilitators to adherence
2018	Fischer N., Agboola S., Palacholla R., Atif M., Jethwani K. & Kvedar J. (2018). A 2-arm randomized pilot study to evaluate the impact of a mobile health application on medication adherence in patients on oral anti-cancer medications. Value in Health, 21(Supplement 1), S35. Retrieved from http://ovidsp.ovid.com/ovidweb.cgi?T=JS&PAGE=reference&D=emed19&NEWS=N&AN=623583696.	• Adherence: Electronic pill bottle<br>• Adherence: Self-report via the Morisky Medication Adherence Scale (MMAS)
2018	Middendorff G., Elsey R., Lounsbery B. & Chadwell R. (2018). Impact of a specialty pharmacy case management service on adherence in patients receiving oral antineoplastic agents. Journal of Oncology Pharmacy Practice, 24(5), 371-378. https://doi.org/10.1177/1078155217708022	• Adherence: MPR

2018	Morgan, K. P., Muluneh, B., Deal, A. M., & Amerine, L. B. (2018). Impact of an integrated oral chemotherapy program on patient adherence. Journal of oncology pharmacy practice : official publication of the International Society of Oncology Pharmacy Practitioners, 24(5), 332–336. https://doi-org.proxy3.library.mcgill.ca/10.1177/1078155217703792	• Adherence: MPR
2018	Muluneh B., Schneider M., Faso A., Amerine L., Daniels R., Crisp B., et al (2018). Improved Adherence Rates and Clinical Outcomes of an Integrated, Closed-Loop, Pharmacist-Led Oral Chemotherapy Management Program. Journal of Oncology Practice, 14(6), e324-e334. https://doi.org/10.1200/JOP.17.00039	• Molecular response rates<br>• Pharmacist intervention<br>• Patient and provider satisfaction<br>• Patient understanding of treatment
2018	Newman V. (2018). Structured patient education utilizing the medication oral agent teaching tool for oncology patients prescribed oral chemotherapy in the outpatient setting. Supportive Care in Cancer, 26(Supplement 3), S393. https://doi.org/10.1007/s00520-018-4356-1	• Adherence: Patient diary<br>• Knowledge: Adherence Starts with Knowledge-12 survey (ASK-12)
2018	Pavese I., Collon T., Chait Y., Cherait A., Bisseux L., MBarek B., et al (2018). Impact on treatment adherence, side effects control, patients QoL, and rehospitalization rate through new management of oral chemotherapy. Journal of Clinical Oncology, 36(15 Supplement 1), no pagination. https://doi.org/10.1200/JCO.2018.36.15-suppl.e18533	• Adherence<br>• QoL<br>• Toxicity<br>• Hospitalization rates
2018	Riu, G., Gaba, L., Victoria, I., Molas, G., do Pazo, F., Gómez, B., Creus, N., & Vidal, L. (2018). Implementation of a pharmaceutical care programme for patients receiving new molecular-targeted agents in a clinical trial unit. European journal of cancer care, 27(1), 10.1111/ecc.12447. https://doi-org.proxy3.library.mcgill.ca/10.1111/ecc.12447	• Adherence: MPR<br>• Pharmaceutical interventions
2018	Sikorskii, A., Given, C. W., Given, B. A., Vachon, E., Krauss, J. C., Rosenzweig, M., McCorkle, R., Champion, V. L., Banik, A., & Majumder, A. (2018). An Automated Intervention Did Not Improve Adherence to Oral Oncolytic Agents While Managing Symptoms: Results From a Two-Arm Randomized Controlled Trial. Journal of pain and symptom management, 56(5), 727–735. https://doi-org.proxy3.library.mcgill.ca/10.1016/j.jpainsymman.2018.07.021	• Adherence: Pill counts<br>• Adherence: RDI<br>• Symptoms
2017	Battis, B., Clifford, L., Huq, M., Pejoro, E., & Mambourg, S. (2017). The impacts of a pharmacist-managed outpatient clinic and chemotherapy-directed electronic order sets for monitoring oral chemotherapy. Journal of oncology pharmacy practice : official publication of the International Society of Oncology Pharmacy Practitioners, 23(8), 582–590. https://doi-org.proxy3.library.mcgill.ca/10.1177/1078155216672314	• Adherence: Refill history<br>• Cognitive impairment<br>• Complexity of regimen<br>• Depression<br>• Missed appointments<br>• Social support<br>• Side effects
2017	Dolezal M.V., Leong V. & Behl R. (2017). Monitoring oral anti-cancer therapy adherence in cancer patients using web based application guided communication compared with nurse counseling in a community cancer center. Journal of Clinical Oncology, 35(5 Supplement 1), no pagination. Retrieved from http://ovidsp.ovid.com/ovidweb.cgi?T=JS&PAGE=reference&D=emed18&NEWS=N&AN=618046957	• Quality of Life: RAND-36
2017	Kimura, M., Go, M., Iwai, M., Usami, E., Teramachi, H., & Yoshimura, T. (2017). Evaluation of the role and usefulness of a pharmacist outpatient service for patients undergoing monotherapy with oral anti-cancer agents. Journal of oncology pharmacy practice : official publication of the International Society of Oncology Pharmacy Practitioners, 23(6), 413–421. https://doi-org.proxy3.library.mcgill.ca/10.1177/1078155216655473	• Adherence to prescription recommendations: Self-report via questionnaire

2017	Mackler E.R., Beekman K.W., Bushey L., Gentz A., Davis K., Yarrington C., et al (2017). Utilizing patient reported outcomes for patients recieving oral chemotherapy. Journal of Clinical Oncology, 35(8 Supplement 1), no pagination. Retrieved from http://ovidsp.ovid.com/ovidweb.cgi?T=JS&PAGE=reference&D=emed18&NEWS=N&AN=618100222.	% PROS complete<br>Side effects<br>Adherence<br>• Self-report
2017	Northcutt L., Drenning J., Ping C.B. & Milks K. (2017). Evaluation of a customized compliance program for oral oncolytic therapy for leukemia. Value in Health, 20(5), A311. Retrieved from http://ovidsp.ovid.com/ovidweb.cgi?T=JS&PAGE=reference&D=emed18&NEWS=N&AN=617599882	Adherence<br>• MPR
2017	Pereira-Salgado, A., Westwood, J. A., Russell, L., Ugalde, A., Ortlepp, B., Seymour, J. F., Butow, P., Cavedon, L., Ong, K., Aranda, S., Breen, S., Kirsa, S., Dunlevie, A., & Schofield, P. (2017). Mobile Health Intervention to Increase Oral Cancer Therapy Adherence in Patients With Chronic Myeloid Leukemia (The REMIND System): Clinical Feasibility and Acceptability Assessment. JMIR mHealth and uHealth, 5(12), e184. https://doi-org.proxy3.library.mcgill.ca/10.2196/mhealth.8349	• Clinical feasibility and acceptability
2017	Rodriguez, G., Utate, M. A., Joseph, G., & St Victor, T. (2017). Oral Chemotherapy Adherence: A Novel Nursing Intervention Using an Electronic Health Record Workflow. Clinical journal of oncology nursing, 21(2), 165–167. https://doi-org.proxy3.library.mcgill.ca/10.1188/17.CJON.165-167	• Adherence: Self-report asked by nurse and documented in system
2017	Tokdemir, G., & Kav, S. (2017). The Effect of Structured Education to Patients Receiving Oral Agents for Cancer Treatment on Medication Adherence and Self-efficacy. Asia-Pacific journal of oncology nursing, 4(4), 290–298. https://doi-org.proxy3.library.mcgill.ca/10.4103/apjon.apjon_35_17	• Medication Adherence Self-Efficacy Scale (MASES)<br>• Symptoms: MSAS
2017	Vella J., Wirth F., Anastasi A., Azzopardi L.M. & Serracino-Inglott A. (2017). Pharmacist-led adherence clinics for Hodgkin's and non-Hodgkin's lymphoma. International Journal of Clinical Pharmacy, 39(1), 307. https://doi.org/10.1007/s11096-016-0404-4	• Adherence: Self-report via the Morisky 8-item Medication Adherence Scale (MMAS-8)
2016	Bellomo, C. (2016). Oral Chemotherapy: Patient Education and Nursing Intervention. Journal of Oncology Navigation & Survivorship . Jul2016, Vol. 7 Issue 6, p20-27. 8p.	• Adherence knowledge: Self-report via Adherence knowledge (ASL-12)
2016	Bourmaud, A., Rousset, V., Regnier-Denois, V., Collard, O., Jacquin, J. P., Merrouche, Y., Lapoirie, J., Tinquaut, F., Lataillade, L., & Chauvin, F. (2016). Improving Adherence to Adjuvant Endocrine Therapy in Breast Cancer Through a Therapeutic Educational Approach: A Feasibility Study. Oncology nursing forum, 43(3), E94–E103. https://doi-org.proxy3.library.mcgill.ca/10.1188/16.ONF.E94-E103	• Anxiety<br>• Knowledge<br>• Trust in the treatment
2016	Degui E., Vinson C., Pelagatti V.C., Canonge J.-M. & Puisset F. (2016). Pharmacist counselling for outpatient treated by oral chemotherapy. International Journal of Clinical Pharmacy, 38(5), 1338. https://doi.org/10.1007/s11096-016-0347-9.	• Number of HCP communicated with
2016	Deutsch, S., Koerner, P., Miller, R. T., Craft, Z., & Fancher, K. (2016). Utilization patterns for oral oncology medications in a specialty pharmacy cycle management program. Journal of oncology pharmacy practice : official publication of the International Society of Oncology Pharmacy Practitioners, 22(1), 68–75. https://doi-org.proxy3.library.mcgill.ca/10.1177/1078155214547664	• Adverse events<br>• Medication discontinuations

2016	Griffiths T. & Pascoe E. (2016). Evaluation of an education program to facilitate patient adherence, toxicity monitoring and promote safety and well-being in the self administration of oral chemotherapy. Supportive Care in Cancer, 24(1 Supplement 1), S78. https://doi.org/10.1007/s00520-016-3209-z	• Patient knowledge and understanding
2016	Jean E.P., Selloum N.E., Regnier O., Poirot B., Abdelghani M.B. & Prebay D. (2016). Pharmaceutical care consultations and multidisciplinary educational program for improving adherence in oncology. International Journal of Clinical Pharmacy, 38(5), 1343. https://doi.org/10.1007/s11096-016-0347-	• Adherence: Self-report via Morisky Green scoring
2016	Lam, M. S., & Cheung, N. (2016). Impact of oncology pharmacist-managed oral anticancer therapy in patients with chronic myelogenous leukemia. Journal of oncology pharmacy practice : official publication of the International Society of Oncology Pharmacy Practitioners, 22(6), 741–748. https://doi-org.proxy3.library.mcgill.ca/10.1177/1078155215608523	Primary:<br>• Adherence: MPR<br>Secondary:<br>• Adverse event<br>• Drug interactions<br>• Dose adjustment<br>• Other drugs taken<br>• Laboratory follow-up
2016	McNamara, E., Redoutey, L., Mackler, E., Severson, J. A., Petersen, L., & Mahmood, T. (2016). Improving Oral Oncolytic Patient Self-Management. Journal of oncology practice, 12(9), e864–e869. https://doi-org.proxy3.library.mcgill.ca/10.1200/JOP.2016.011304	• Documentation in chart
2016	Murugan K., Ostwal V., Carvalho M.D., D'souza A., Achrekar M.S., Govindarajan S., et al (2016). Self-identification and management of hand-foot syndrome (HFS): effect of a structured teaching program on patients receiving capecitabine-based chemotherapy for colon cancer. Supportive Care in Cancer, 24(6), 2575-2581. https://doi.org/10.1007/s00520-015-3061-6	• Patient knowledge
2016	Page R.D., Conerly N., Ward L. & Hodges A. (2016). Novel management of oral chemotherapy adherence using Navigating Cancer's patient-reported outcomes mobile application. Journal of Clinical Oncology, 34(Supplement 15), no pagination. Retrieved from http://ovidsp.ovid.com/ovidweb.cgi?T=JS&PAGE=reference&D=emed17&NEWS=N&AN=611756150.	• Adherence: Self-report via app<br>• Symptoms and adverse events
2016	Patel, J. M., Holle, L. M., Clement, J. M., Bunz, T., Niemann, C., & Chamberlin, K. W. (2016). Impact of a pharmacist-led oral chemotherapy-monitoring program in patients with metastatic castrate-resistant prostate cancer. Journal of oncology pharmacy practice : official publication of the International Society of Oncology Pharmacy Practitioners, 22(6), 777–783. https://doi-org.proxy3.library.mcgill.ca/10.1177/1078155215612541	• Number of interventions<br>• Overall time on therapy
2016	Rasschaert M., Helsen S., Rolfo C., Van Brussel I., Ravelingien J. & Peeters M. (2016). Feasibility of an interactive electronic self-report tool for oral cancer therapy in an outpatient setting. Supportive Care in Cancer, 24(8), 3567-3571. https://doi.org/10.1007/s00520-016-3186-2	• Adherence: Self-report via app<br>• Toxicity
2016	Ribed, A., Romero-Jiménez, R. M., Escudero-Vilaplana, V., Iglesias-Peinado, I., Herranz-Alonso, A., Codina, C., & Sanjurjo-Sáez, M. (2016). Pharmaceutical care program for onco-hematologic outpatients: safety, efficiency and patient satisfaction. International journal of clinical pharmacy, 38(2), 280–288. https://doi-org.proxy3.library.mcgill.ca/10.1007/s11096-015-0235-8	Primary:<br>• Adverse events<br>• Drug-related problems<br>• Drug interactions<br>Secondary:<br>• Adherence (MPR)<br>• Patient satisfaction<br>• Permanence

2016	Todo M., Arakawa I., Ueda S., Osaki A. & Saeki T. (2016). Collaborative pharmacotherapy involving physicians and pharmacists aimed at improving adherence in everolimus therapy for advanced/recurrent breast cancer and its outcomes. Supportive Care in Cancer, 24(1 Supplement 1), S108. https://doi.org/10.1007/s00520-016-3209-z	• QoL<br>• Medical costs
2016	Todo M., Arakawa I., Ueda S., Osaki A. & Saeki T. (2016). Collaborative pharmacotherapy involving physicians and pharmacists aimed at improving adherence in everolimus therapy for advanced/recurrent breast cancer and its outcomes. Supportive Care in Cancer, 24(1 Supplement 1), S108. https://doi.org/10.1007/s00520-016-3209-z	Primary:<br>• Medication persistence: Medical record<br>• Interventions<br>Secondary:<br>• Adverse events<br>• Cost<br>• Drug interactions
2016	Wong, S. F., Bounthavong, M., Nguyen, C. P., & Chen, T. (2016). Outcome Assessments and Cost Avoidance of an Oral Chemotherapy Management Clinic. Journal of the National Comprehensive Cancer Network : JNCCN, 14(3), 279–285. https://doi-org.proxy3.library.mcgill.ca/10.6004/jnccn.2016.0033	• Medication persistence: Medical record<br>• Types and outcomes of interventions
2016	Zhao I., Lai J., Seike B., Wilson M., Brennan L., Li T., et al (2016). Evaluation of a pharmacist-driven oral chemotherapy adherence program. Journal of Oncology Pharmacy Practice, 22(2 Supplement 1), 3. https://doi.org/10.1177/1078155215624650	Adherence:<br>MPR
2015	Boucher, J., Lucca, J., Hooper, C., Pedulla, L., & Berry, D. L. (2015). A Structured Nursing Intervention to Address Oral Chemotherapy Adherence in Patients With Non-Small Cell Lung Cancer. Oncology nursing forum, 42(4), 383–389. https://doi-org.proxy3.library.mcgill.ca/10.1188/15.ONF.383-389	• Adherence: Self-report via the Morisky Medication Adherence Scale–8 (MMAS-8)<br>• Knowledge: Knowledge Rating Scale (KRS)
2015	Hendricks C. B. (2015). Improving adherence with oral antiemetic agents in patients with breast cancer receiving chemotherapy. Journal of oncology practice, 11(3), 216–218. https://doi-org.proxy3.library.mcgill.ca/10.1200/JOP.2015.004234	• Adherence: Medical record
2015	Ramesh A., Rajanandh M.G., Thanmayee S., Merin G.S., Suresh S. & Srinivas K.S. (2015). Impact of patient counseling on medication adherence, beliefs and satisfaction about oral chemotherapies in patients with metastatic cancer at a super specialty hospital. International Journal of Cancer Research, 11(3), 128-135. https://doi.org/10.3923/ijcr.2015.128.135	• Adherence: Self-report via MARS<br>• Belief about Medication Questionnaire (BMQ)
2015	Sheridan B., Larmour I., Manser A., Sturm S., Allan B., Fazli O., et al (2015). Compliance enhancing patient information leaflets-a strategy to decrease patient misadventure with oral chemotherapies. Pharmacotherapy, 35(11), e250-e251. https://doi.org/10.1002/phar.1659	• Patient perceived usefulness
2015	Spoelstra, S. L., Given, C. W., Sikorskii, A., Coursaris, C. K., Majumder, A., DeKoekkoek, T., Schueller, M., & Given, B. A. (2015). Feasibility of a Text Messaging Intervention to Promote Self-Management for Patients Prescribed Oral Anticancer Agents. Oncology nursing forum, 42(6), 647–657. https://doi-org.proxy3.library.mcgill.ca/10.1188/15.ONF.647-657	• Adherence: RDI<br>• Symptoms: Symptom Experience Inventor
2014	Bordonaro, S., Romano, F., Lanteri, E., Cappuccio, F., Indorato, R., Butera, A., D'Angelo, A., Ferraù, F., & Tralongo, P. (2014). Effect of a structured, active, home-based cancer-treatment program for the management of patients on oral chemotherapy. Patient preference and adherence, 8, 917–923. https://doi-org.proxy3.library.mcgill.ca/10.2147/PPA.S62666	• Adherence: Self-report via questionnaire<br>• Quality of Life: EORTC QLQ-C30
2014	Campbell, C. (2014). Nursing intervention to improve adherence and safety with oral cancer therapy. Canadian Oncology Nursing Journal, 24(4), 302-309.	• Patient knowledge<br>• Chart review: Adverse events, number of cycles, and dose reductions

2014	Clottens N. (2014). Integration of a clinical pharmacist in a multidisciplinary team for renal cell carcinoma patients treated with oral chemotherapy: Impact on adherence and financial aspects. Journal of Oncology Pharmacy Practice, 20(3 SUPPL. 1), 23-24. https://doi.org/10.1177/1078155214523700	• Adherence: Refills<br>• Financial cost
2014	Schneider, S. M., Adams, D. B., & Gosselin, T. (2014). A tailored nurse coaching intervention for oral chemotherapy adherence. Journal of the advanced practitioner in oncology, 5(3), 163–172.	• Adherence: Pharmacy refill rates<br>• Adherence: Self-report via questionnaire
2014	Wong, S. F., Bounthavong, M., Nguyen, C., Bechtoldt, K., & Hernandez, E. (2014). Implementation and preliminary outcomes of a comprehensive oral chemotherapy management clinic. American journal of health-system pharmacy : AJHP : official journal of the American Society of Health-System Pharmacists, 71(11), 960–965. https://doi-org.proxy3.library.mcgill.ca/10.2146/ajhp130278	• Adherence: Self-report by patient, assessed on phone<br>• Adverse events<br>• Drug interactions<br>• Medication errors<br>• Symptom management
2013	Gebbia V., Bellavia M., Banna G.L., Russo P., Ferrau F., Tralongo P., et al (2013). Treatment monitoring program for implementation of adherence to second-line erlotinib for advanced non-small-cell lung cancer. Clinical Lung Cancer, 14(4), 390-398. https://doi.org/10.1016/j.cllc.2012.11.007	• Adherence: Pill count<br>• Adherence: Self-report via Basel Assessment of Adherence Scale (BAAS)
2013	Spoelstra, S. L., Given, B. A., Given, C. W., Grant, M., Sikorskii, A., You, M., & Decker, V. (2013). An intervention to improve adherence and management of symptoms for patients prescribed oral chemotherapy agents: an exploratory study. Cancer nursing, 36(1), 18–28. https://doi-org.proxy3.library.mcgill.ca/10.1097/NCC.0b013e3182551587	• Adherence: Pharmacy refill<br>• Adherence: Self-report<br>• Symptom: Symptom Experience Inventory
2012	Bordonaro, S., Raiti, F., Di Mari, A., Lopiano, C., Romano, F., Pumo, V., Giuliano, S. R., Iacono, M., Lanteri, E., Puzzo, E., Spada, S., & Tralongo, P. (2012). Active home-based cancer treatment. Journal of multidisciplinary healthcare, 5, 137–143. https://doi-org.proxy3.library.mcgill.ca/10.2147/JMDH.S31494	• Adherence: Self-report via questionnaire<br>• Healthcare utilization<br>• QoL: EORTC QLQ-C30
2012	Khandelwal, N., Duncan, I., Ahmed, T., Rubinstein, E., & Pegus, C. (2012). Oral chemotherapy program improves adherence and reduces medication wastage and hospital admissions. Journal of the National Comprehensive Cancer Network : JNCCN, 10(5), 618–625. https://doi-org.proxy3.library.mcgill.ca/10.6004/jnccn.2012.0063	• Adherence: MPE<br>• Side effects and adverse events
2012	Smith K. (2012). The development and evaluation of an oncology patient compliance tool. Journal of Oncology Pharmacy Practice, 18(SUPPL. 2), 11. https://doi.org/10.1177/1078155212439813	• Patient satisfaction and perceptions of program
2012	Sommers R.M., Miller K. & Berry D.L. (2012). Feasibility pilot on medication adherence and knowledge in ambulatory patients with gastrointestinal cancer. Oncology Nursing Forum, 39(4), E373-E379. https://doi.org/10.1188/12.ONF.E373-E379	• Adherence: Self-report via MMAS-8
2012	Wang Y. (2012). An analysis of using pamphlets of oral chemotherapy guidelines and calendars for patients less educated. Supportive Care in Cancer, 20(SUPPL. 1), S81. https://doi.org/10.1007/s00520-012-1479-7	• Adherence: Self-report via MMAS-8
2012	Welslau M., Haase S., Jakob A. & Marschner N. (2012). Quality assurance of oral Xeloda chemotherapy by a mobile phone application. Journal of Cancer Research and Clinical Oncology, 138(SUPPL. 1), 33. https://doi.org/10.1007/s00432-011-1144-4	• Adherence/compliance: Self-report on App

2011	Samuel L.M., Lynch D., Christie G., Collie J., McLachlan N., Jordan J., et al (2011). Nurse-lead clinics for capecitabine: A prospective audit evaluating changes in toxicity profile and efficacy. Journal of Clinical Oncology, 29(4 SUPPL. 1), no pagination. Retrieved from http://ovidsp.ovid.com/ovidweb.cgi?T=JS&PAGE=reference&D=emed12&NEWS=N&AN=70679774.	• Adverse events<br>• Dose reductions
2011	Simons S., Ringsdorf S., Braun M., Mey U.J., Schwindt P.F., Ko Y.D., et al (2011). Enhancing adherence to capecitabine chemotherapy by means of multidisciplinary pharmaceutical care. Supportive Care in Cancer, 19(7), 1009-1018. https://doi.org/10.1007/s00520-010-0927-5	• Adherence: Electronic pill cap (MEMS)
2010	Oakley, C. Johnson & Ream, E.. (2010). Developing an intervention for cancer patients prescribed oral chemotherapy: a generic patient diary. European Journal of Cancer Care19, 21–28	• Adherence self-efficacy: Cancer Behavior Inventory Brief Form (CBI-B)<br>• Patient perceptions<br>• Symptom management: Memorial Symptom Assessment Scale Short Form (MSAS-SF)
